# Editorial: The microbial modulation of autoimmune processes and proinflammatory pathways

**DOI:** 10.3389/fcimb.2022.969610

**Published:** 2022-08-02

**Authors:** Elias A. Rahal, Margret Shirinian, Daniele Dessì

**Affiliations:** ^1^ Department of Experimental Pathology, Immunology and Microbiology, American University of Beirut, Beirut, Lebanon; ^2^ Center for Infectious Disease Research, American University of Beirut, Beirut, Lebanon; ^3^ Dipartimento di Scienze Biomediche, Università Degli Studi di Sassari, Sassari, Italy

**Keywords:** dysbiosis, inflammation, autoimmune diseases, microbiome, infection

Microbial agents, whether they are part of the human microbiome or extrinsic organisms, can modulate immune processes, potentially resulting in exacerbated inflammatory responses or their amelioration; microbial products can also play similar roles. This may favor or curb the initiation of a proinflammatory disorder or alter the course of an ongoing disease. Mirroring the effect of microbial agents on modulation of immune responses, an inflammatory disease may shape an individual’s microbiome, resulting in modification of the disease state (Belkaid and Hand, 2014). Hence, a two-way street exists between the organisms we harbor and our immune processes. With the Research Topic “*The Microbial Modulation of Autoimmune Processes and Proinflammatory Pathways*,” we aimed at examining the various facets of how microbial organisms and inflammatory conditions affect each other. The Research Topic comprises eight papers that investigate the roles played by members of the microbiota in modulating inflammation and vice versa, pathways that play a role in these processes, alterations in immune processes induced by microbial products, and the prognostic value of particular markers of inflammation in certain infections.

The study by Yu et al. examines the fecal microbiome of rheumatoid arthritis (RA) patients and controls using 16S rDNA sequencing. The authors also used liquid chromatography–tandem mass spectrometry (LC-MS/MS) to conduct metabolic profiling of the fecal samples. While previous studies have focused on the roles that butyrate and short-chain fatty acids may play in decreasing the severity of arthritis (Rosser et al., 2020; Martinsson et al., 2022), this is the first study to systematically examine the full spectrum of changes in the gut flora as well as the metabolomic profile in RA. The study reveals alterations in the abundances of several genera and in the levels of a number of metabolites; data indicate that dysbiosis and dysregulation of several pathways, including those of the metabolism of tryptophan, alpha-linolenic acid, and glycerophospholipid, may play a significant role in the development of RA. This may highlight novel biomarkers as well as therapeutic avenues for this disease.

On the other hand, Toyofuku et al. studied the effect of haploinsufficiency of A20 (HA20) on the gut microbiome of affected subjects. HA20 is a relatively recently described autoinflammatory disorder resulting from dysregulation of NF-kB signaling (Aeschlimann et al., 2018). Not only did the study included in the Research Topic at hand show dysbiosis in HA20 patients, the severity of the disease was also associated with a lower number of operational taxonomic units (OTUs). Moreover, the study revealed that *Streptococcus mutans* and *Lactobacillus salivarius* may increase in patients with autoantibodies compared with those who do not have such autoantibodies. The implications of these finding on the management or prognosis of HA20 require further investigations.

The study by Khadka et al. demonstrates that administration of curcumin monoglucuronide (CMG) to the experimental autoimmune encephalomyelitis (EAE) mouse model of multiple sclerosis modulated the disease state. Moreover, this treatment altered the fecal and ileal microbiota. A correlation between the severity of EAE and the ileal microbiota was also noted. The administration of curdlan to the same mouse model by Sato et al. worsened the disease condition, resulting in enhanced central nervous system infiltration and increased proinflammatory cytokine levels. In contrast, administration of curdlan to the viral mouse model of MS, the Theiler’s murine encephalomyelitis virus-induced demyelinating disease (TMEV-IDD) model, ameliorated the disease severity. These studies may indicate that the specific underlying cause of a disease should be considered when developing a management plan. The implication here is that two patients with the same inflammatory disorder may not respond in a similar manner depending on the disease triggering or exacerbating conditions.

In exploring the roles ASB17 plays in immune processes, Wan et al. observed that it mediates NF-kB signal activation and subsequent cytokine production; this occurs *via* stabilizing TRAF6 by inhibiting the latter’s ubiquitination. The indicated observations were made in bone marrow–derived dendritic cells (BMDCs) stimulated with lipopolysaccharide (LPS). ASB17 is an ankyrin repeat and SOCS box-containing protein (ASB) family member whose biological roles are still under investigation; hence, this study sheds light on this mediator, revealing its relevant role in the regulation of cytokine production in response to microbial products. The immunomodulatory effects of another microbial product, Shiga toxin type 2a (Stx2a), were examined by Rosso et al. This bacterial toxin is usually produced by particular strains of *Escherichia coli* that can result in bloody diarrhea and hemolytic uremic syndrome (HUS), a kidney-affecting, potentially fatal disorder (Joseph et al., 2020). In the study by Rosso et al., Stx2a-stimulated human glomerular endothelial cells activated γδ T cells and induced the production of proinflammatory cytokines by these T cells as well as favored their differentiation toward a Th1-like profile. This is the first report to describe a possible role for human γδ T cells in the pathogenesis of Shiga toxin–associated HUS.

On the other hand, a study by Zheng et al. shows that higher blood D-dimer (D-d) levels correlate with disease severity in children with *Mycoplasma pneumoniae* pneumonia (MPP). D-d is a fibrinolysis product that is often correlated with inflammatory conditions (Ala et al., 2019; Ge et al., 2019; Zhang et al., 2021; Feng et al., 2022). Not only did the levels of D-d correlate with the length of hospital stay and the duration of fever in these patients, levels of this marker also correlated with those of inflammatory mediators, such as IL-6 and IFN-γ. On the other hand, the study by Qi et al. demonstrates that higher blood levels of acetic acid correlate with mortality in *Pseudomonas aeruginosa* ventilator-associated pneumonia. Increased acetic acid levels were also associated with decreased blood levels of lymphocytes and monocytes; this may provide some mechanistic insight as to how acetic acid results in a poor prognosis, which merits further exploration. Hence, both of these studies provide novel prognostic markers that may reflect the inflammatory status as well as the severity of particular types of pneumonia.

Overall, the collection of papers in this Research Topic highlight various novel means by which microbes or their products and immune responses shape each other. Such studies often suggest intervention modes that may prove useful in the alleviation of inflammatory disease conditions triggered or exacerbated by microbial agents.

## Author contributions

All authors listed have made a substantial, direct, and intellectual contribution to the work and approved it for publication.

## Funding

ER is supported by grants from the American University of Beirut-Medical Practice Plan (MPP), the Lebanese National Council for Scientific Research (L-CNRS) and the Asmar Fund. MS is funded by the American University of Beirut-Medical Practice Plan (MPP), the Lebanese Council for Scientific Research (CNRS) and the King Hussein Cancer Research Award. DD is supported by University of Sassari, Grant FAR 2020.

## Acknowledgments

We would like to thank the authors of the papers included in this Research Topic as well as the reviewers who contributed to assessing these studies. We also wish to thank the Frontiers editorial team, particularly members of the Frontiers in Cellular and Infection Microbiology editorial office, for their hard work.

## Conflict of interest

The authors declare that the research was conducted in the absence of any commercial or financial relationships that could be construed as a potential conflict of interest.

## Publisher’s note

All claims expressed in this article are solely those of the authors and do not necessarily represent those of their affiliated organizations, or those of the publisher, the editors and the reviewers. Any product that may be evaluated in this article, or claim that may be made by its manufacturer, is not guaranteed or endorsed by the publisher.
